# Platelet-derived biomaterials-mediated improvement of bone injury through migratory ability of embryonic fibroblasts: *in vitro* and *in vivo* evidence

**DOI:** 10.18632/aging.202311

**Published:** 2021-01-10

**Authors:** Yen-Ru Chou, Wen-Cheng Lo, Navneet Kumar Dubey, Jui-Hua Lu, Hen-Yu Liu, Ching-Yu Tsai, Yue-Hua Deng, Chi-Ming Wu, Mao-Suan Huang, Win-Ping Deng

**Affiliations:** 1 Graduate Institute of Biomedical Materials and Tissue Engineering, College of Biomedical Engineering, Taipei Medical University, Taipei, Taiwan; 2 School of Medicine, College of Medicine, Taipei Medical University, Taipei, Taiwan; 3 Department of Neurosurgery, Taipei Medical University Hospital, Taipei, Taiwan; 4 School of Dentistry, College of Oral Medicine, Taipei Medical University, Taipei, Taiwan; 5 Stem Cell Research Center, College of Oral Medicine, Taipei Medical University, Taipei, Taiwan; 6 Department of Dentistry, Taipei Medical University-Shuang Ho Hospital, New Taipei, Taiwan; 7 Graduate Institute of Biomedical and Pharmaceutical Science, Fu Jen Catholic University, Taipei, Taiwan; 8 Department of Life Science, Tunghai University, Taichung, Taiwan

**Keywords:** platelet-derived biomaterials, bone injury, TGF-β1, fibroblasts migration, osteogenesis

## Abstract

Bony injuries lead to compromised skeletal functional ability which further increase in aging population due to decreased bone mineral density. Therefore, we aimed to investigate the therapeutic potential of platelet-derived biomaterials (PDB) against bone injury. Specifically, we assessed the impact of PDB on osteo-inductive characteristics and migration of mouse embryonic fibroblasts (MEFs). Osteogenic lineage, matrix mineralization and cell migration were determined by gene markers (RUNX2, OPN and OCN), alizarin Red S staining, and migration markers (FAK, pFAK and Src) and EMT markers, respectively. The therapeutic impact of TGF-β1, a key component of PDB, was confirmed by employing inhibitor of TGF-β receptor I (Ti). Molecular imaging-based *in vivo* cellular migration in mice was determined by establishing bone injury at right femurs. Results showed that PDB markedly increased expression of osteogenic markers, matrix mineralization, migration and EMT markers, revealing higher osteogenic and migratory potential of PDB-treated MEFs. *In vivo* cell migration was manifested by expression of migratory factors, SDF-1 and CXCR4. Compared to control, PDB-treated mice exhibited higher bone density and volume. Ti treatment inhibited both migration and osteogenic potential of MEFs, affirming impact of TGF-β1. Collectively, our study clearly indicated PDB-rescued bone injury through enhancing migratory potential of MEFs and osteogenesis.

## INTRODUCTION

Bone is a highly specialized and dynamic tissue which provides shape, mechanical support and facilitates body movement. Additionally, bone also contributes to mineral homeostasis of body and has recently been demonstrated to regulate energy metabolism [1, 2]. With the increase in aging population, the incidence of bone-associated trauma such as injury, fracture or osteoporosis will inevitably rise among women and men. This could be attributed to decreased bone mineral density (BMD) resulting in remarkable alterations in bone material and its structural aberration. Following bone injury, the cellular migration and differentiation, tissue synthesis, and release of growth factor/ cytokine occurs, under guidance of mechanical environment. Further, the geometry, microarchitecture and size of bone are key parameters impacting the ability to withstand trauma, and about 75%–90% of bone strength is associated with BMD [3]. It is approximated that 5-10% of bone fractures/injuries results in disunion or late healing and hence remain a key management in orthopedic surgery [4].

The current surgical approaches for treating large bony defect reconstruction employ autologous bone segments harvest, which leads to associated morbidity and complications and prolonged hospitalization thereby increasing direct/indirect healthcare burden. Therefore, it is highly imperative to find novel healing therapies for bone injury prevention [5]. Fibroblasts are the prominent cells in wound healing phenomenon, as they migrate and proliferate to the injury site and produce extracellular matrix (ECM) proteins to form connective tissue [6]; therefore participate in their self-renewal, tissue repair, facilitate the functional integrity of the various organs. These cells have widely been employed in the form of feeder layers to assist the growth and expansion of embryonic stem cells; of which the most commonly are mouse embryonic fibroblasts (MEFs) [7]. Both the MEFs as well as mesenchymal stem cells share common characteristics such as plastic adherence, identical morphological appearance surface markers, and multipotent nature [8, 9]. Though bone healing is not yet completely understood, the researches have indicated that various growth factors may influence reparative process and healing response of therapeutic cells like MEFs. Recent studies including ours have also shown that platelet-derived biomaterials (PDB) containing a plethora of growth factors could accelerate wound healing, suppress inflammation and regenerate tissues such as kidney, brain and bone regeneration [10–12]. After activation or physical disruption of the platelet α-granule structure, the therapeutic effects are mediated through release factors such as platelet-derived growth factor (PDGF), vascular endothelial growth factor (VEGF), platelet-derived angiogenesis factor (PDAF), epidermal growth factor (EGF), transforming growth factor-β (TGF-β), and insulin-like growth factor (IGF)-1 [13, 14]; of which TGF-β1 is the key growth factor and multifunctional cytokine that governs various biological processes, such as cellular differentiation, apoptosis, immunity, and the production of the ECM [15]. To corroborate the TGF-β1 mediated therapeutic impact, we also used inhibitor of TGF-β receptor I (Ti), which specifically bind to TGF-β1 receptor.

**Figure 1:**
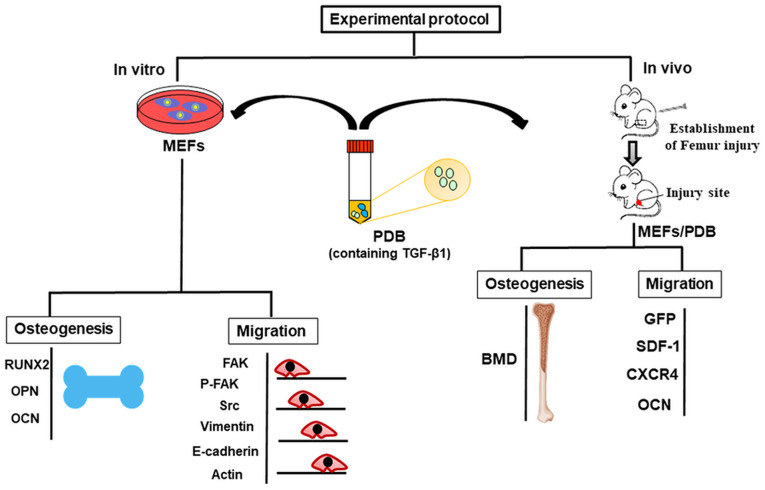
**Schematic of *in vitro* and *in vivo* experimental protocol representing migration and osteogenic potential of platelet-derived biomaterials (PDB) containing TGF-β-1 on mouse embryonic fibroblasts (MEFs) to improve bone injury.** During *in vitro* assay, the osteogenic potential was evaluated through expression of markers RUNX2, OPN, and OCN; whereas, migratory ability was determined by expression levels of FAK, p-FAK, Scr, Vimentin, and E-cadherin. Further, after PDB treatment of bone injury in FVB mice, the bone mineral density (BMD) of femur was noted, while cellular migration was assessed via SDF-1, GFP, OCN to evaluate bone injury improvement.

Furthermore, PDB is safe and biocompatible due to biological origin, hence the chances of immune response or infection could be avoided. Therefore, in this study, we investigated the impact of PDB-contained TGF-β1 on proliferative, migratory and osteo-inductive characteristics of mouse embryonic fibroblasts (MEFs) in bone injury mice model, as described in experimental protocol (Figure 1).

## RESULTS

### Impact of PDB on *in vitro* osteogenesis and migration of mouse embryonic fibroblasts (MEFs)

**Figure 2:**
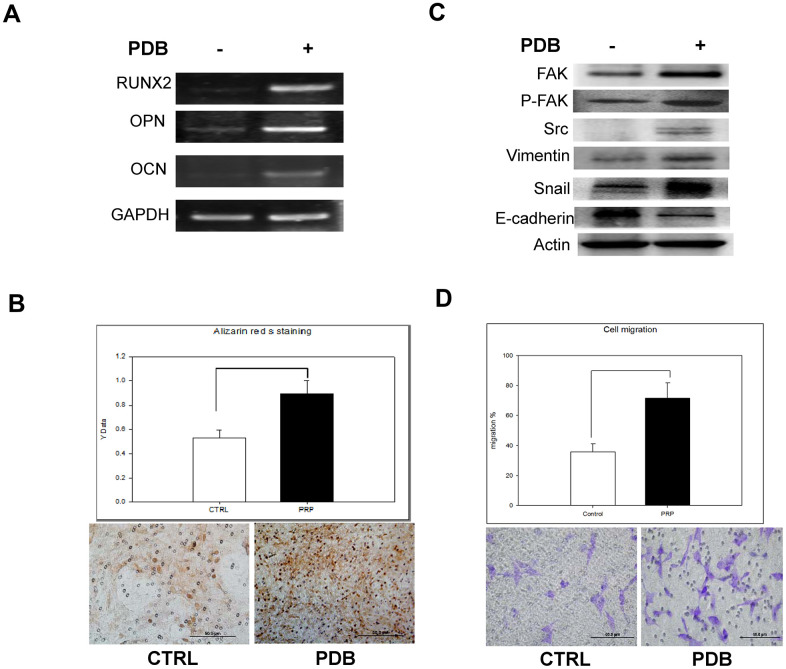
***In vitro* analysis of osteoblastic differentiation and cell migration of osteoblasts treated with PDB.** (**A**) RT-PCR analysis dependent expression of osteogenic markers (RUNX2, OCN, and OPN). (**B**) Quantitative analysis of matrix mineralization in MEFs by alizarin Red S staining. (**C**) Protein expression of migratory and epithelial to mesenchymal transition (EMT) markers potential of MEFs. (**D**) Quantification analysis of transwell cell migration assay. The experimental group represent PDB-treatment; whereas, no any treatment served as control. The representative results of 3 experiments demonstrated mean ± SD. *P < 0.05.

To investigate the *in vitro* effect of PDB on osteogenesis, MEFs were cultured in media with or without PDB for 21 days. We found that PDB-treated group revealed a marked increase in gene expression of osteogenic markers RUNX 2, OPN and OCN (Figure 2A). Subsequently, we used Alizarin Red S staining on the MEFs to determine degree of mineralization, which was also enhanced in PDB-treated group (Figure 2B, lower panel). The quantified results also demonstrated a 2-fold higher staining intensity when compared to the CTRL group (Figure 2B, higher panel).

Further, we examined the migratory and epithelial to mesenchymal transition (EMT) markers potential of MEF_S_. Our data showed an enhanced protein levels of migration-associated markers (FAK, pFAK and Src) as well as EMT markers (vimentin and snail) in PDB-treated MEFs; whereas, E-cadherin was found to be suppressed when compared to the CTRL group (Figure 2C). After a 48 hours’ incubation period, transwell cell migration assay showed increased number of migrated cells in PDB-treated MEFs (Figure 2D, lower panel). These results were further confirmed through quantification of staining showing enhanced ratio of migrated cells in PDB-treated MEFs (Figure 2D, upper panel).

### Effect of PDB on MEFs migratory potential to bone injury site

**Figure 3:**
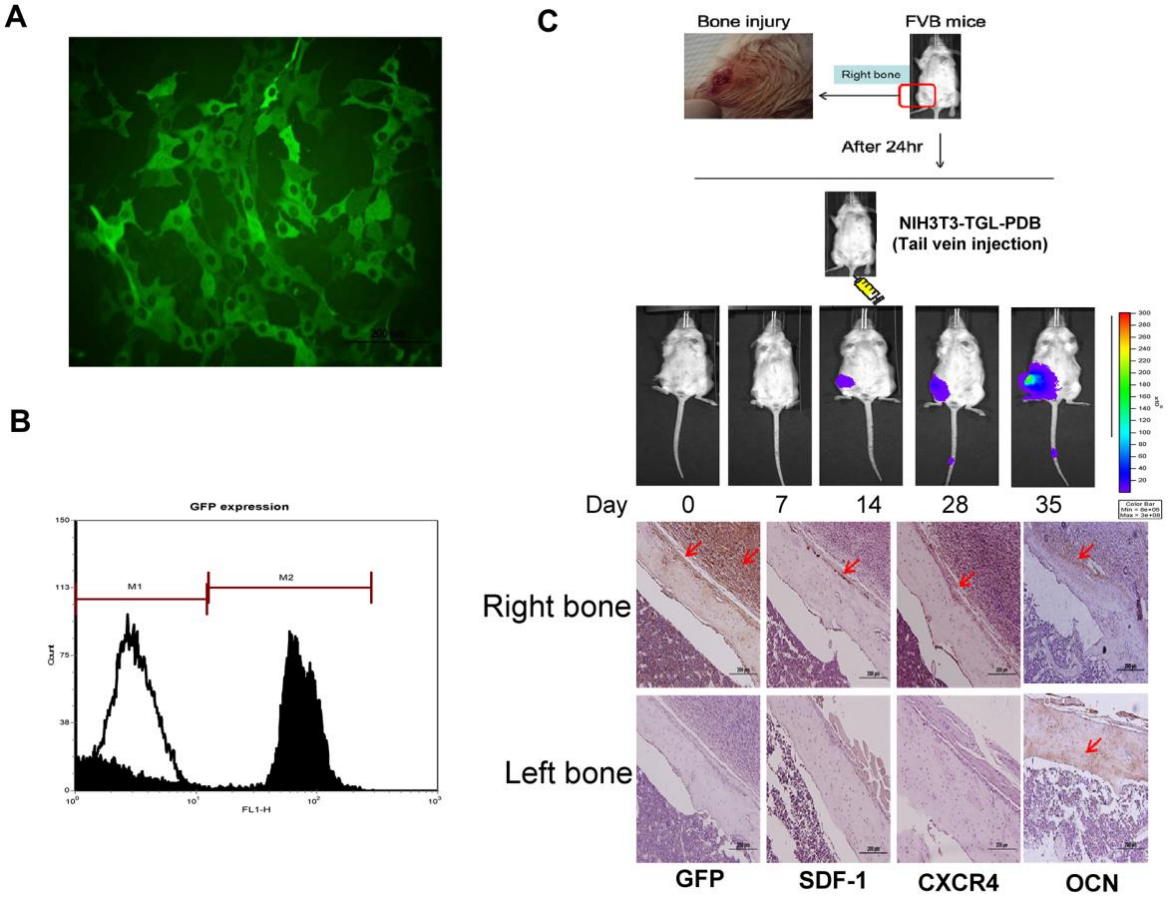
***In vitro* characterization and *in vivo* therapeutic effect of GFP transfected MEFs (NIH3T3) containing lentivirally transduced genes, thymidine kinase (T), green fluorescence protein (G) and luciferase genes (L) (NIH3T3-TGL).** (**A**) GFP transfected NIH3T3 cell line were sorted out among other cells types by flow cytometry and the expression of GFP in MEF-TGL cells was validated by fluorescent microscopy exhibiting strong green signals (**B**). (**C**) Experimental presentation of femur bone injury created at mid-diaphysis of bilateral right femur, and cellular migration from administered site in FVB mice. Increased GFP-positive cells were detected at indicated time points. Histologic images also confirm the presence of GFP-positive signals (brown color) in right femur, while no signal appeared in left femur (control).

Based on increased expressions of migration and EMT markers, we then investigated the PDB effect on cell migration *in* *vivo* by molecular imaging through establishing bone injury at right femurs. For optical imaging, we also established MEF-TGL cell line through lentiviral transduction of thymidine kinase (T), green fluorescence protein (G) and luciferase genes (L) into MEFs. The expression of GFP in MEF-TGL cells was validated by fluorescent microscopic (Figure 3A) and flow cytometry (Figure 3B).

After 24 hours, we transplanted PDB-treated MEF-TGL through tail vein injection and observed the cellular migration by optical imaging. After 14 days, stronger signals were observed at the injury site, which were further increased at 28 and 35 days (Figure 3C, upper panel). Immunohistochemical analysis revealed an increase in GFP-positive cells in the newly formed bone. We also found that PDB-treated MEFs had migrated to the injury site, which was evidenced through expression of migratory factors, SDF-1 and CXCR4. Further, the osteogenic potential was evidenced by increased expression of osteogenic markers OCN after 35 days of transplantation (Figure 3C, lower panel).

### Corroborating proliferative and migratory regulation of PDB-contained TGF-β through TGF-β receptor I inhibitor (Ti) activity

**Figure 4:**
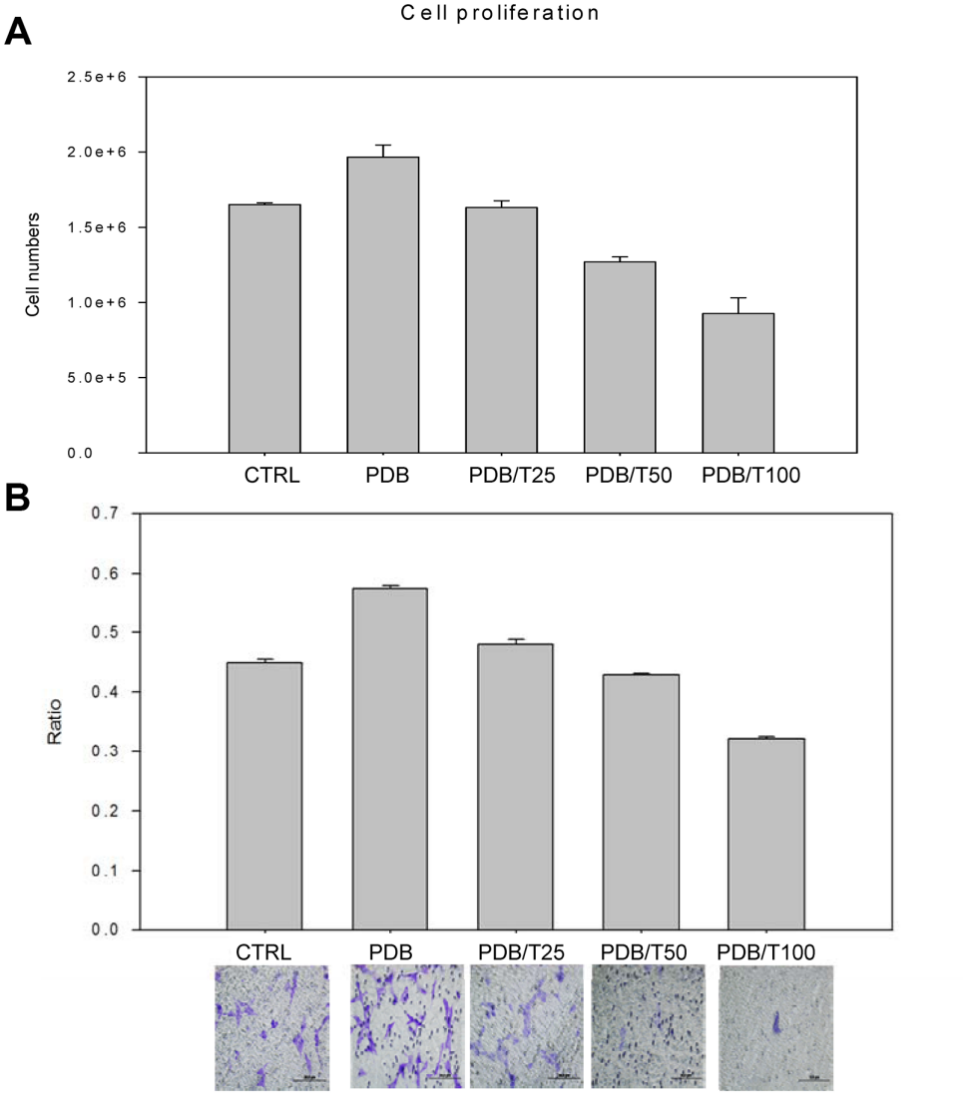
**Optimization of concentration of inhibitor of TGF-β receptor I (Ti).** The growth and migratory regulation through Ti dose-dependent (25-100nM) assay of (**A**) Cellular proliferation and (**B**) Transwell cell migration and its relative quantification revealed degenerative characteristics when compared to control; whereas, the PDB showed a stimulatory effect. The representative results of 3 experiments demonstrated mean ± SD.

Based on the above-mentioned evidences for strong efficacy of PDB on MEFs migratory potential, we further confirmed its role by using Ti, which specifically binds to TGF-β. The TGF-β has been recognized as a key component among the plethora of PDB [12] promoting cellular migration [16]. We first optimized Ti inhibitory concentration, which showed the degenerative characteristics in terms of cellular proliferation in a dose-dependent fashion (Figure 4A), when compared to control; whereas, the PDB showed a stimulatory effect. This result was also consistent in transwell cell migration assay and its quantification revealing decreased number of migrated cells in Ti-treated cells (Figure 4B). These above results imply that Ti suppressed cellular migration through inhibiting TGF-β1; whereas, the PDB could stimulate the cellular growth.

### PDB-mediated regulation of cell migratory migration through Ti activity

**Figure 5:**
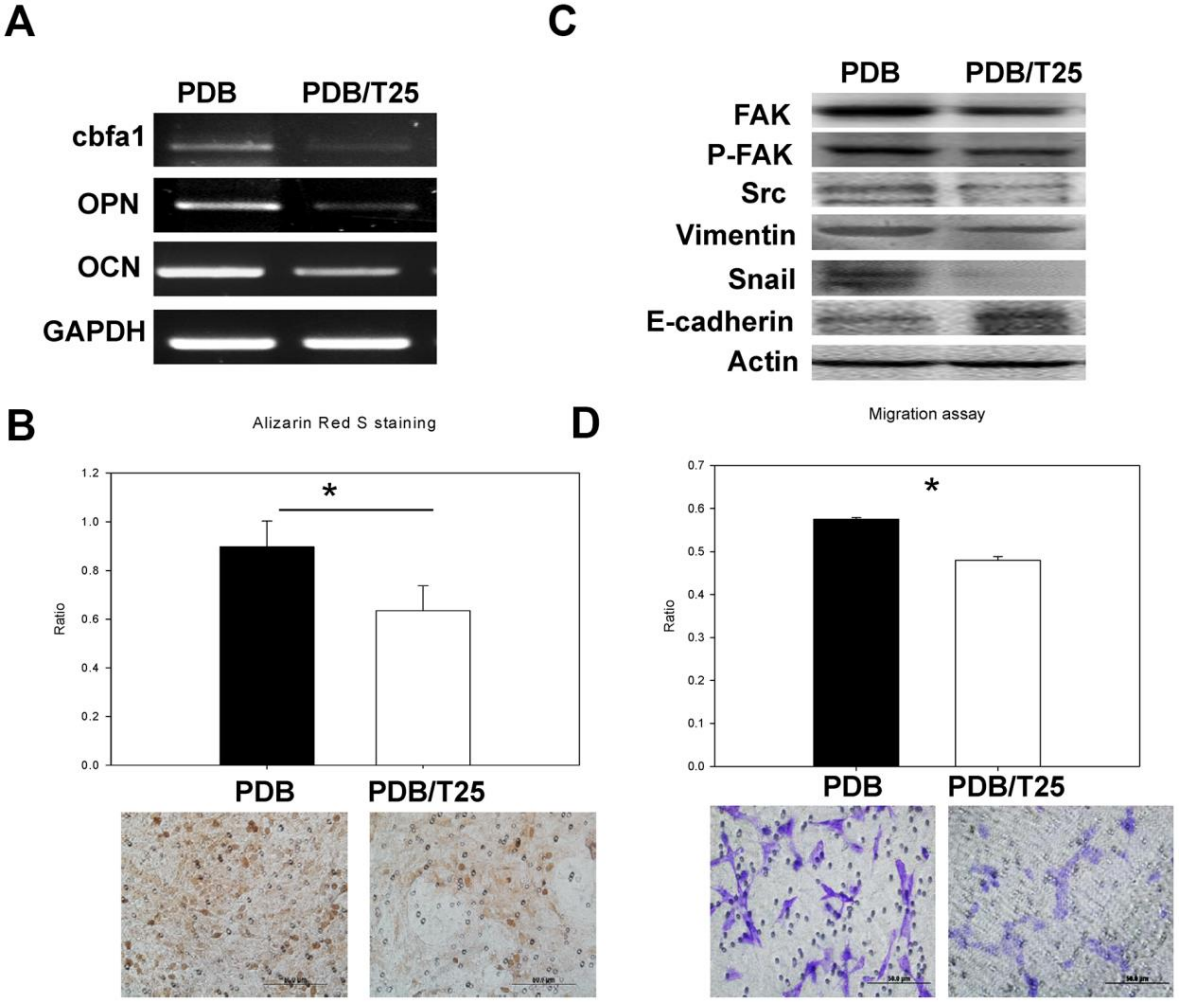
***In vitro* analysis of osteoblastic differentiation and cell migration of osteoblasts treated by PDB with and without inhibitor of TGF-β receptor I (Ti) in a concentration of 25nM (Ti25).** (**A**) RT-PCR analysis dependent expression of osteogenic markers (RUNX2, OCN, and OPN). (**B**) Quantitative analysis of matrix mineralization in MEFs by alizarin Red S staining. (**C**) Protein expression of migratory and epithelial to mesenchymal transition (EMT) markers potential of MEFs. (**D**) Quantification analysis of transwell cell migration assay. The representative results of 3 experiments demonstrated mean ± SD. *P < 0.05.

We further examined PDB-induced migratory potential of MEFs by Ti through mRNA levels of migratory factor (RUNX2) in Ti-treated MEFs, which was found inhibited, indicating TGF-β1 stimulatory activity towards MEFs migration (Figure 5A). Ti treatment also demonstrated a decreased gene expression of osteogenic markers OPN, and OCN when compared to PDB. Subsequently, we used Alizarin Red S staining on the MEFs to determine degree of mineralization with Ti or PDB treatment. The results revealed markedly reduced staining intensity in Ti-treated cells (Figure 5B, lower panel). Quantitatively, this decrease was found to be 1.5-fold lower compared to PDB group (Figure 5B, upper panel). Further, the Ti or PDB-treated MEFs were investigated for their cellular migration through determining the levels of migratory (FAK, pFAK and Src) and EMT (vimentin, E-cadherin, and snail) markers. We found that Ti significantly reduced all migration markers and EMT markers; whereas, the expression of E-cadherin was increased when compared to PDB group (Figure 5C). Besides, after 48 hours’ incubation period, the Ti-treated MEFs showed decreased number of migrated cells by staining during transwell migration assay (Figure 5D, lower Panel). These results were further confirmed through quantified staining, which also showed decreased ratio of migrated cells in Ti-treated MEFs (Figure 5D, upper panel).

### Effect of PDB on *in vivo* osteogenesis

**Figure 6:**
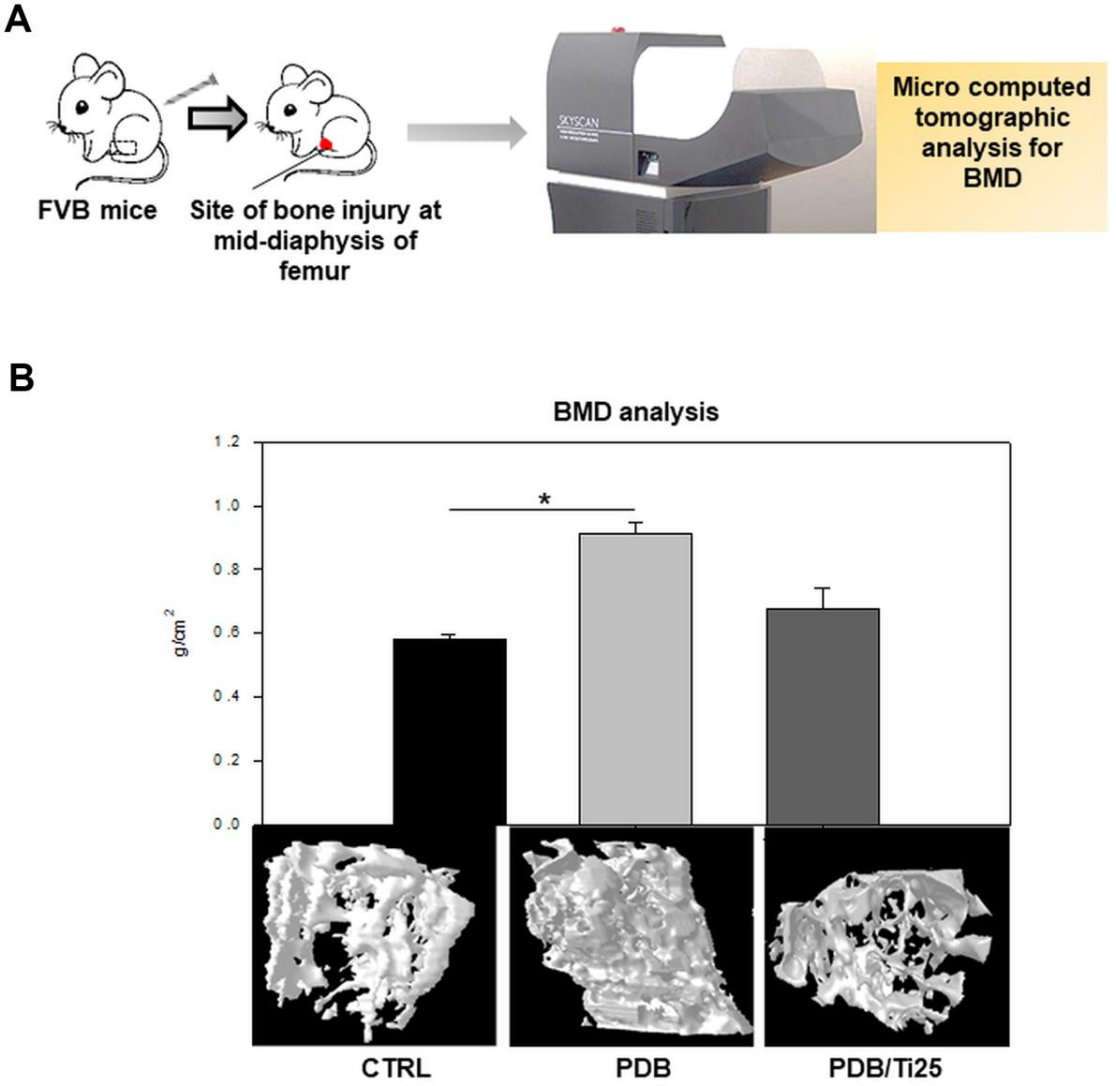
***In vivo* analysis of bone mineral density (BMD).** (**A**) Schematic describing microcomputed tomographic (μCT) analysis of BMD in FVB mice, and (**B**) 3D photomicrographs of femurs from control, PDB and PDB/Ti25 treated group mice, and their quantitative of bone density. The experimental groups represent PDB and PDB+Ti25 treatment; whereas, no any treatment served as control. *P < 0.05.

To investigate *in vivo* osteogenic potential, FVB mice were intravenously injected with PDB or Ti-treated MEFs for 35 days (Figure 3C), and bone injury structural sites at mid-diaphysis of bilateral right femur were analyzed through μCT, as described in the schematic (Figure 6A). We found that the group transplanted with PDB-treated MEFs exhibited an increased bone density and volume when compared to the CTRL group (Figure 6B). In contrast, no significant difference between Ti and CTRL group was found. These outcomes clearly indicated that PDB rescued from bone injury by enhancing *in vivo* osteogenesis.

## DISCUSSION

**Figure 7:**
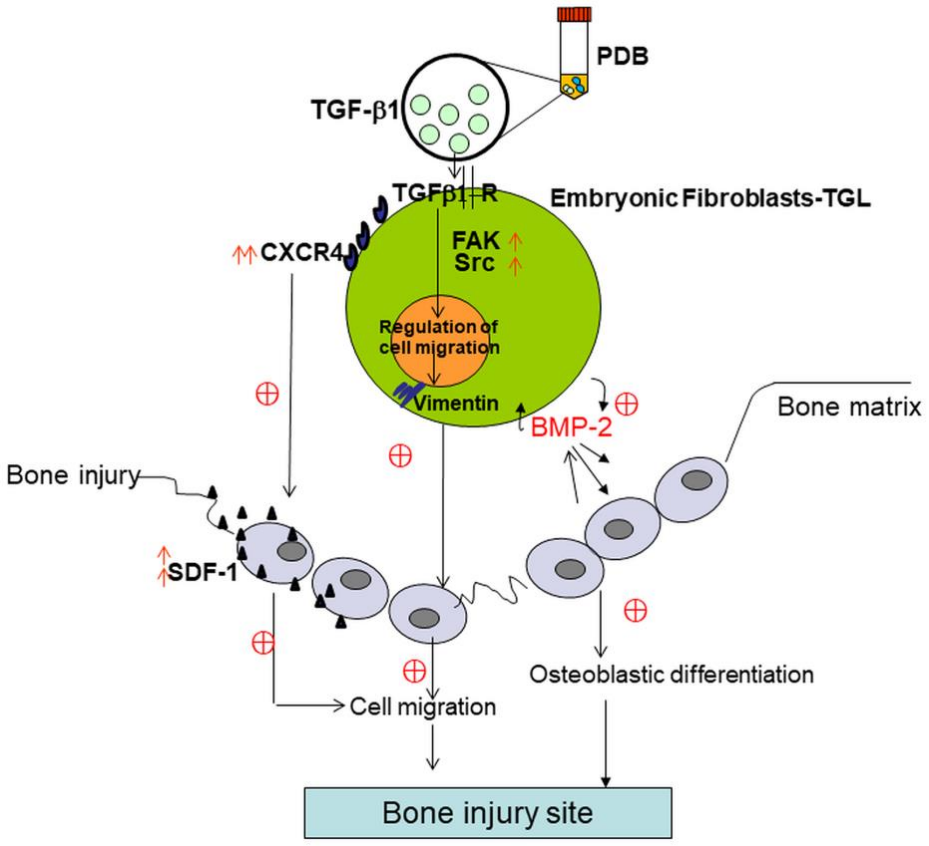
**Schematic of possible mechanistic insight into PDB-mediated improvement of bone injury through osteogenic and migratory ability of embryonic fibroblasts.**

Though a body of evidence exist on beneficial effects of PDB on various pathologies at the molecular, cellular, animal, and human levels; till date, there has been no reliable evidence on the efficacy of PDB treatment for bone injury. However, in present study, the PDB accelerated healing of bone injury, could be mediated possibly by osteogenesis and migration of mouse embryonic fibroblasts (MEFs) (Figure 7). During wound healing process, the fibroblasts play a crucial role, which around the injured region differentiate into myofibroblasts, a type of highly contractile cells that produce abundant extracellular matrix (ECM) proteins [17]. It is also noted that cell migration is also a key factor for bone synthesis and treatment of bony disorders [18]. While addressing the influence of PDB, we found a prominent increase in expression of osteogenic markers, degree of mineralization, migration and EMT markers. These results are supported through a study by Chen et al. showing significant healing of skin excisional wounds in rats by nerve growth factor (NGF)-mediated by fibroblast migration [19]. Another report also suggested skin wound closure by trophic activity of mesenchymal stem cells which influence both dermal fibroblast and keratinocyte migration to the synthesize ECM [20]. During osteogenesis, it has been evidenced that vascular endothelium migrates and synthesize a network of small capillaries to supply oxygen and nutrients to newly formed tissues [21].

These therapeutic impacts of PDB might be attributed to TGF-β1, a key cytokine contained in PDB [12]. TGF-β have been reported to support the formation of fibrosis after muscular injury by stimulating the production of extracellular matrix proteins and inhibiting their degradation, thereby normalize muscle tissue [22]. After confirming the PDB-mediated enhanced migration of MEFs *in vitro*, we then examined its efficacy on cell migration *in* *vivo* by molecular imaging through generating bone injury at right femurs. The effect was observed in the terms of stronger and increasing fluorescence signals of transplanted PDB-treated MEF-TGL and enhanced GFP-immunopositive cells at injury site and newly formed bone, respectively. Studies have revealed that SDF-1/CXCR4 axis is highly important for bone marrow stem cells migration to the injury sites under various pathologies, including spinal cord lesions, myocardial infarction and cerebral ischemia [23–25]. SDF-1 mediated cell homing signals play a key role in its mobilization to the injured microenvironment and are therefore crucial for improving bone repair [24]. Based on these evidences, the expressed SDF-1 and CXCR4 in our PDB-treated MEFs clearly indicated their migration and homing to bone injury site, where increased levels of OCN implied new bone synthesis after 35 days of transplantation.

To further confirm TGF-β1-induced migration of MEFs, we employed inhibitor of TGF-β receptor I (Ti) specifically binding to TGF-β1 receptor and determined its inhibitory concentration, the dose of 25nM of which inhibited cellular proliferation and migration. Thus, it could be inferred that during coupled processes of recruiting MEFs to bone remodeling surfaces and osteogenesis, the TGF-β1 functions as a primary factor, and its dysregulation leads to pathological state. Further, the RUNX2 is a multifunctional transcription factor and an essential master gene controlling osteoblast differentiation and enhances their migration by coupling with PI3K-Akt signaling [26, 27]. While examining PDB-induced migratory potential of MEFs in the presence of Ti, the levels of RUNX2 found inhibited, implying TGF-β1 stimulatory activity towards MEFs migration. During osteogenesis, OPN and OCN are well-known intermediate and late stage markers of osteoblasts [28]. In comparison to PDB, the Ti also suppressed gene expression of OPN and OCN, suggesting TGF-β1 promoted osteogenesis. This was further supported by alizarin Red S staining for determining calcium deposits stained as dark red regions [29] which appeared feeble in Ti-treated group when compared to PDB.

Cellular migration is a concerted phenomenon which needs continuous, coordinated assembly and disassembly of adhesion structures. During migration, focal adhesion kinase (FAK) plays in signaling cascade initiated through interacting between integrins and ECM proteins [30, 31] and results in phosphorylation of tyrosine 397 (Y397) of FAK, which promotes the process. It has also been noted that fibroblasts lacking FAK are unable to migrate in response to normal stimuli. However, both Src (a non-receptor tyrosine kinase) and FAK bind, phosphorylate, and stimulate exchanging of factors (guanine nucleotide exchange factors) of Rho-family small GTPases regulating cytoskeleton organization and initiating cell migration [32]. Besides, the EMT is a process in which the cellular epithelial characteristics changed to interstitial under specific physiological or pathophysiological conditions [33]. In our study, while the occurrence of EMT, the expression of E-cadherin, (major marker adhering between the epitheliums), was downregulated, whereas, the vimentin (the major interstitial marker), was upregulated leading to reduced adhesive forces among cells of PDB treated-group, which led to increased cell migration. Finally, during *in vivo* osteogenic activity analysis, the 3D micro-CT imaging data of PDB-treated mice revealed an enhanced trabecular bone synthesis and hence the bone mineral density (BMD) and decreased bone loss compared to Ti-treated group. BMD has been shown to be an important factor to determine the strength of cancellous bone and trabecular bone [28, 34] *in vivo*. These outcomes implied that PDB could retard the bone loss and improve bone recovery. The effectiveness of PDB could also be ascribed to the anti-inflammatory activities of TGF-β1 [35] and lipoxins [36], modulating repair of inflamed tissues leading to bony matrix restoration [37] and regeneration [38]. Furthermore, the other PDB-contained growth factors in the cocktail which may participate in therapeutic outcomes include platelet-derived growth factor (PDGF) epidermal growth factor (EGF), hepatocyte growth factor (HGF) and basic fibroblast growth factor (bFGF) [39]. It has been evidenced that PDGF-BB may organize the presence of osteoprogenitor cells at a requisite site, stimulate their efficient replication, regulate their responsiveness to osteoblastic differentiation factors and stabilize the newly synthesized blood vessels [40]. Besides, EGF and bFGF are powerful mitogens for various types of cells including MSCs [41, 42] The EGF not only promotes cell proliferation but also increase matrix mineralization [43]; whereas, bFGF enhances activation of osteoblast differentiation and osteogenesis [44]. Previous studies have also revealed the synergy between HGF and VEGF, which may likely to act in coherence in normal physiology [45, 46], implying a role in non-inflammatory instead of inflammatory angiogenesis [47, 48]. Hence, it is possible that PDB could modify the angiogenic balance through triggering the secretion of HGF [49]. Besides several significant therapeutic outcomes, our study also includes a few limitations, such as confirming roles of other specific PDB-contained biomaterials on improvement of bone injury through migratory ability of embryonic fibroblasts. In addition, detailed underlying therapeutic mechanism will also be investigated.

Taken together, our results indicate the role of PDB contained TGF-β1 in enhancing embryonic fibroblasts migratory ability to improve bone injury and also deduced the possible underlying therapeutic mechanism. During repair and regeneration of bone injury, the administered PDB-treated MEFs reversed the pathologic characteristics primarily by enhancing migratory factors and BMD leading to osteogenesis (Figure 7).

## MATERIALS AND METHODS

### Cell line and their culture

The mouse NIH/3T3 cells (mouse embryonic fibroblast, ATCC CRL 1658) were obtained from the American Type Culture Collection were procured from Bioresource Collection and Research Centre and maintained under conditions described by the ATCC. Further, using Lipofectamine 2000 system (Invitrogen). These cells were genetically modified to stably express the enhanced GFP reporter gene (NIH3T3-G cells), which were further expanded and enriched to over 0.95% by fluorescence-activated cell sorting.

### Preparation of platelet-derived biomaterials (PDB)

The PDB (TGF-β1=1ng/ml) was prepared by protocols as described previously [12]. Briefly, we employed the specialized platelet concentrate separator containing ACD-A as anticoagulant and a specific separator gel that harvest platelets and plasma preventing contamination of other blood components including red blood cells (RBC) and leukocytes. Human peripheral blood (7 mL) was collected into a PLTenus PLUS Platelet Concentrate Separator (TCM Biotech International Corp., Taiwan) via a sterile venipuncture. Thereafter, the obtained blood was centrifuged at 500–1200G for 8 min and mixed plasma and platelets (4 mL) remaining above the gel layer, collected in a falcon tube for future use.

### RNA extraction and semi-quantitative reverse-transcription PCR

Total RNA was isolated using the TRIzol® reagent (Life Technologies) and subjected to reverse transcription (RT) using RevertAid H minus first strand cDNA synthesis kit (Thermo scientific). RT product was used for PCR amplification with GoTaq® Green Master Mix (Promega). PCR products were electrophoresed in 2% agarose gels (AMRESCO) with SYBR safe (BIOTOOLS Co.) staining, and images were examined through Mutigel-21 (Fluorescent Gel Image System TOP BIO Co.). The PCR primers were as follow: Runt-related transcription factor 2 (RUNX2) -forward primer forward primer 5’-ACTTTCTCCAGGAAGACTGC-3’; reverse primer 5’-GCTGTTGTTGCTGTTGCTGT-3’; temperature 55° C.; Osteopontin (OPN)-forward primer 5’-ATGAGATTGGCAGTGATT-3’; reverse primer 5-GTTGACCTCAGAAGATGA-3’; temperature, 48.8° C; Osteocalcin (OCN)-forward primer 5’-CAGCTTGGTGCACACCTAAGC-3’; reverse primer 5’- AGGGTTAAGCTCACACTG-3’; temperature, 55° C; glyceraldehyde 3 phosphate dehydrogenase (GAPDH) which was used as an internal control (CTRL) forward primer 5’-GCTCTCCAGAACATCATCCCTGCC-3’; reverse primer 5’-CGTTGTCATACCAGGAAATGACTT-3’; temperature 55° C.

### Confirmation of TGF-β1 signaling through TGF-β receptor I inhibitor (Ti) activity

To confirm the PDB-contained TGF-β1 signaling, the MEFs were treated with Ti (SB-505124, Sigma-Aldrich, 100 nM).

### Cell migration assay

A total of 1 × 10^5^ fibroblast cells were seeded into the upper Transwell chamber (6.5 mm diameter with a pore size of 8 mm; Corning, Inc.) while in the lower chamber, 8mL Dulbecco's Modified Eagle's Medium, with 1% bovine calf serum was added and the assay was done for 24 h at 37° C and 5% CO_2_. At indicated time points, cells were fixed with 3% formaldehyde and filters were stained with the hematoxylin (Sigma, Inc). Cells that had not migrated from the upper side of the filters were scraped off with a cotton swab. The number of cells that had migrated to lower side of the filter were counted under a light microscope with five high-power fields (200X). Experiments were done in triplicate.

### Animal studies and ethics

All animal studies were approved by the Institutional Animal Care and Use Committee of Taipei Medical University, Taiwan. Six week-old female FVB mice were purchased from National Laboratory Animal Center, Taipei, Taiwan. The mice were kept under pathogen-free conditions and fed autoclaved food and water. For the experimental purpose, the mice were divided into 3 groups: control (n=6), PDB (n=6) and PDB+T25 group (n=6). Both PDB and T25 were injected with a microsyringe into the femur.

### Creation of femoral bone defect and assessment of bone mineral density (BMD)

At day 0, a transcortical hole was drilled using a dental bur at mid-diaphysis of bilateral femurs, producing a bone defect of 1.5 mm in diameter. The mice each from the control, PDB and PDB+T25 group were sacrificed at day 35 after injury. Bilateral femoral samples were obtained by cutting along the long axis of the femur including the mid region of drilled site at the anterior cortex. The BMD of femurs was assessed by dual-energy x-ray absorptiometry. This test was initially performed at time 0 (at 0.5 mo) and once at 35 days for different treatment regimes (the endpoint of the experiment was set at 3 mo after the ovariectomy). The BMD of femurs was measured and collected using a densitometer (XR-36; Norland Corp.; host software revision 2.5.3, scanner software revision 2.0.0).

### Immunohistochemistry and western blot analysis

Bone sections (10 mm thick) from the femur of mice were collected, washed, and fixed using 10% paraformaldehyde solution in phosphate-buffered saline at room temperature. All primary antibodies used were purchased from FAK (Cat. no. 3285; Cell Signaling Technology), p-FAK (cat. no. 3284; Cell Signaling Technology), Src (cat. no. 2108; Cell Signaling Technology), vimentin (cat. no. 5741; Cell Signaling Technology) Snail (cat. no. 3879; Cell Signaling Technology), E-cadherin (cat. no. 3195; Cell Signaling Technology), GFP (cat. no. 5385; Cell Signaling Technology). Respective secondary antibodies were used (1:2,000 dilution) and immunoperoxidase reactions were performed using a Vectastain Universal Elite ABC Kit (Vector Laboratories) according to the manufacturer’s protocol. Immunoblotting of control and PRP-treated NIH3T3-G protein extracts was performed according to a standard protocol. Briefly, harvested NIH3T3-G cells were lysed with radioimmunoprecipitation assay (25 mM Tris-HCl, pH 7.6; 150 mM NaCl; 1% NP-40; 1% sodium deoxycholate; and 0.1% sodium dodecylsulfonate (SDS) and extraction buffers (Pierce). Cell lysates were separated by SDS-polyacrylamide gel electrophoresis. Equal amounts of both control and PRP-treated samples were loaded. For Western blotting, proteins from gels were transferred to the polyvinylidene difluoride (PVDF) membrane (pore size, 0.5 mm; Schleicher and Schuellx) using a Mini Trans-Blot Cell apparatus (Bio-Rad Laboratories). Immunodetection reaction was visualized by SuperSignal West Pico Chemiluminescent substrate (Biolynx Inc.).

### Statistical analysis

All results were represented as mean ± standard deviation (SD). All results were represented as mean ± standard deviation (S.D.). Significant differences between the groups were evaluated by student's *t-test* with p value < 0.05.
